# Contraband tobacco on post-secondary campuses in Ontario, Canada: analysis of discarded cigarette butts

**DOI:** 10.1186/1471-2458-13-335

**Published:** 2013-04-11

**Authors:** Meagan Barkans, Kelli-an Lawrance

**Affiliations:** 1 Brock University, Leave The Pack Behind (PL514), 500 Glenridge Avenue, St, Catharines L2S 3A1, ON, Canada; 2 Brock University, Community Health Sciences Department (AS320), 500 Glenridge Avenue, St, Catharines L2S 3A1, ON, Canada

**Keywords:** Contraband tobacco, Young adults, Post-secondary students, Smoking behaviours, Tobacco control strategies, Public health, Unobtrusive observation

## Abstract

**Background:**

No studies to date have assessed young adults’ use of First Nations/Native tobacco, a common form of contraband tobacco in Canada. This study examined the proportion of First Nations/Native cigarette butts discarded on post-secondary campuses in the province of Ontario, and potential differences between colleges and universities and across geographical regions.

**Methods:**

In 2009, discarded cigarette butts were collected from high-traffic smoking locations at 12 universities and 13 colleges purposively selected to represent a variety of institutions from all 7 health service regions across Ontario. Cigarette butts were identified as First Nations/Native tobacco if they were: known First Nations/Native brands; had names not matching domestic and international legally-manufactured cigarettes; had no visible branding or logos.

**Results:**

Of 36,355 butts collected, 14% (95% CI = 9.75–19.04) were First Nations/Native. Use of this tobacco was apparent on all campuses, accounting for as little as 2% to as much as 39% of cigarette consumption at a particular school. Proportions of First Nations/Native butts were not significantly higher on colleges (*M* = 17%) than universities (*M* = 12%), but were significantly higher in the North region.

**Conclusions:**

The presence of cheap First Nations/Native (contraband) tobacco on post-secondary campuses suggests the need for regulation and public education strategies aimed to reduce its use. Strategies should account for regional variations, and convey messages that resonate with young adults. Care must be taken to present fair messages about First Nations/Native tobacco, and avoid positioning regulated tobacco as a healthier option than contraband.

## Background

In the province of Ontario, Canada, 19% of young adults (20 to 24 years old) smoke tobacco [1], and annual surveillance data suggest tobacco use in this age cohort is plateauing after years of decline [2]. Raising the price of tobacco products (through higher taxation) is one of the most effective ways to reduce smoking participation among smokers of all ages but especially younger smokers [3-9]. The apparent widespread availability of inexpensive contraband tobacco in Ontario has been viewed with concern because of its relationship with increased tobacco consumption and decreased quitting intentions [10-13].

Information provided by the Royal Canadian Mounted Police (RCMP) suggests that the types of contraband tobacco most widely available in Canada include: lawfully manufactured, tax-exempt cigarettes designated for purchase by Aboriginals on First Nations Reserves that are illegally diverted to the general population; unlawfully manufactured Canadian products (often produced by Native manufacturers); and, cigarettes produced by Native manufacturers or their counterparts in the U.S. and then smuggled into Canada [14]. Other forms of contraband include international products that have been smuggled into Canada; counterfeit tobacco products; and tobacco products from other criminal activities [14]. Availability of contraband tobacco is not unique to Canada; black markets of untaxed, unregulated, counterfeited, or stolen tobacco products are evident in European [15-17] and south Asian countries [18-21], as well as Australia [22,23] and United States [24,25]. Although sources and distribution channels of illicit tobacco vary, its presence can critically undermine public health strategies to reduce tobacco-related morbidity and mortality, particularly among price-sensitive populations such as youth, low-income earners and socially-deprived groups [3,19,22,26,27].

Evidence that Canadian youth are accessing contraband tobacco comes from a variety of sources. For example, annual studies initiated by the Canadian Convenience Stores Association showed that, in 2007, 24% of the cigarette butts collected from 56 high school properties in two centrally-located, major Ontario cities were identifiable as contraband tobacco [28]. In 2008, with data collection extended to 80 high schools dispersed more widely across Ontario, 26% of the butts collected were identified as contraband [29]. In 2009, the proportion of contraband butts collected from an even wider sample of 110 sites ranged from 19% to 39%, with an overall proportion of 30% [30]. Self-report data from Canada’s annual Youth Smoking Survey have shown that 8% of Ontario high school students who currently smoke [31] and 22% of those who smoke daily [10] “usually” smoke “cigarettes from First Nations / Native brand cigarettes” (i.e., cigarettes meeting those studies’ definitions of contraband tobacco). Furthermore, students who usually smoke contraband cigarettes report significantly higher tobacco consumption rates than those smoking premium name brands [12]. Similar relationships have been observed for adult smokers: usual use of inexpensive tobacco (e.g. contraband or discount cigarettes) is associated with higher rates of consumption and lower intentions to quit [6,11,13,19,22,26,32].

These studies suggest contraband may be undermining tobacco control strategies aimed at preventing tobacco use among youth [4,8,10,12]. It is less clear, however, what impact contraband tobacco is having on young adults’ tobacco use. This is a meaningful question given that initiation and escalation of tobacco use can occur in young adulthood. For example, Hammond [33] reports that approximately 20% of young adults who smoke tried their first cigarette after the age of 18. Similarly, although experimentation with smoking may begin in adolescence, many smokers do not become established, regular smokers until after the age of 18 [33,34]. The availability of cheap contraband tobacco may be contributing to the relatively high prevalence of smoking among young adults by reducing cost barriers that would otherwise inhibit escalation and continuation of tobacco consumption [3,35]. By virtue of having the highest prevalence of smoking among all age groups, young adults have been and continue to be a high priority population for interventions that minimize smoking participation and hasten progression to former smoker status [36]. Learning more about the prevalence of contraband tobacco use among young adults would suggest whether contraband tobacco should be specifically addressed in public health strategies to curb tobacco use in this high-risk population.

The current study explored the extent of young adults’ use of First Nations/Native tobacco through an analysis of discarded cigarette butts on college and university campuses. Given that smuggled brand-name and counterfeit cigarettes represent a very small proportion of contraband tobacco in Ontario [14], while large proportions of First Nations/Native tobacco are produced or consumed illicitly and are reasonably assumed to represent contraband tobacco [10,12,13,31,37], the current study restricted the definition of contraband tobacco to First Nations/Native tobacco cigarette butts. The goals of the study were to: determine the total proportion of First Nations/Native cigarette butts discarded on the grounds of Ontario colleges and universities; and examine possible differences between college and university campuses and among geographical regions of the province.

## Methods

### Data collection

**Figure 1:**
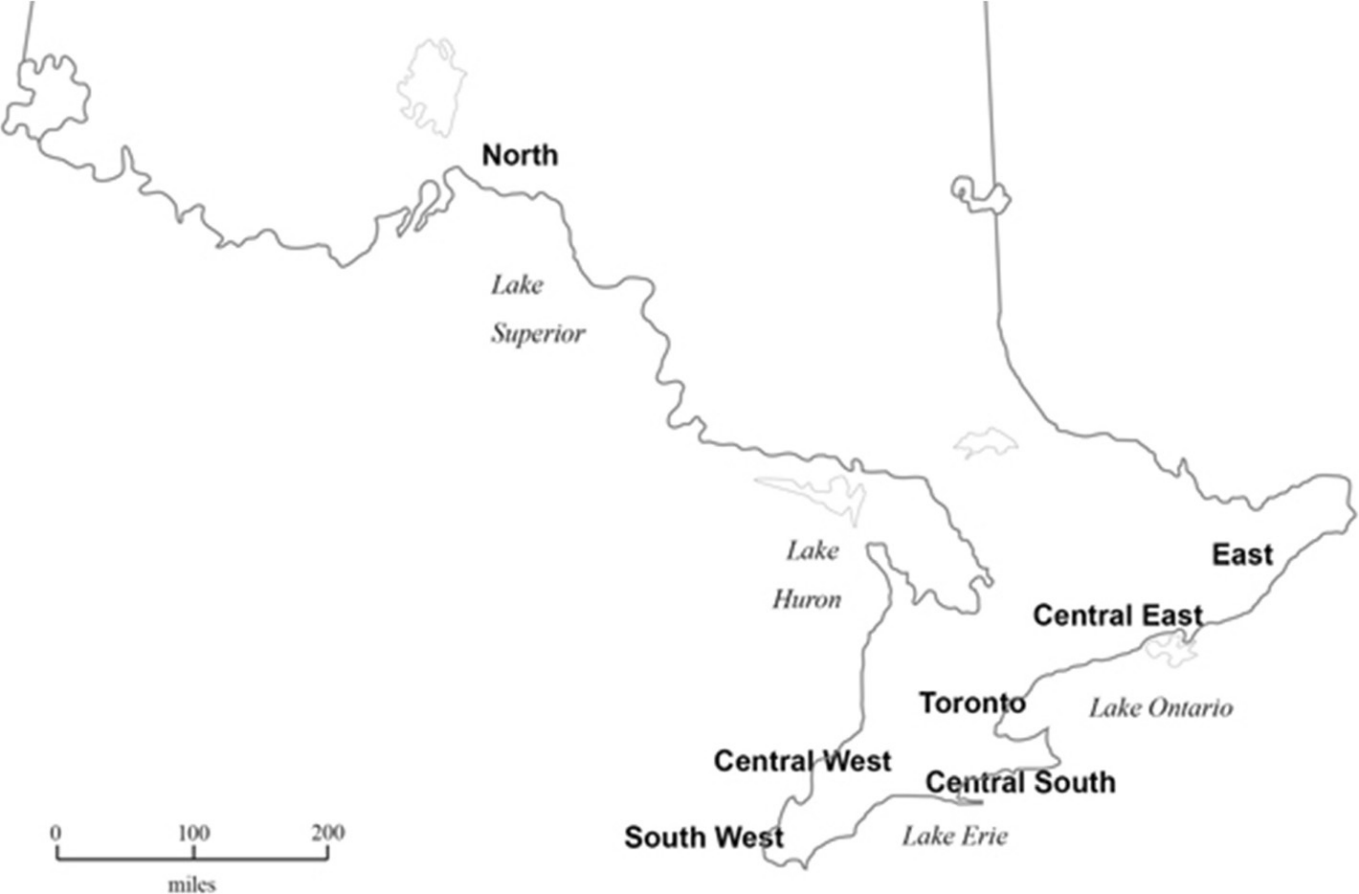
Ontario health service regions.

From the Ontario population of publicly-funded 4-year, degree-granting universities (*N* = 22) and 2-year diploma-granting colleges (*N* = 26), a purposive sample of 12 universities and 13 colleges was selected. At least 2 institutions were selected from each of Ontario’s seven health service regions (shown in Figure 1).

At each institution, trained research assistants collected cigarette butts from four separate smoking areas near: the student centre; a campus pub; a campus residence; and a busy, on-campus bus stop.^a^ If one of these four sites did not exist on the campus, or if smoking did not occur at that site, another high-traffic smoking area used primarily by students was selected based on suggestions from school personnel. These sites represented designated as well as spontaneous smoking areas used primarily by students.

Cigarette butts were collected from any receptacle in the area, and from the ground in an approximate 6-foot radius where the concentration of cigarette butts was heaviest. At each site, collection proceeded until a 2-litre collection container for that site was full, or all discarded butts within the defined area had been collected. All collection occurred between March 20 and April 10, 2009. The day and time of data collection varied from school to school based on the recommendations of school personnel.

Cigarette butts were identified as First Nations/Native tobacco if they were: (1) known Native brands such as *Putter, DK* and *SAGO*; or (2) had brand names that did not match a comprehensive list of domestic and international legally-manufactured cigarettes; or (3) had no visible branding, logos or other defining marks. Cigarettes in the latter two categories were defined as contraband based on an exhaustive internet search of worldwide cigarette brands, information provided by the RCMP (M. Harvey, personal communication, April 27, 2009), and the knowledge that legally-manufactured cigarette brands have visible branding on the filters. Cigarette butts, including those judged to be legally-manufactured domestic and international brands, possible counterfeits, or ‘unidentifiable’ (due to mutilation of the filter tip) were classified as non-Native tobacco. Categorization of cigarettes was done by two raters, and settled by a third rater in the very infrequent cases where the first two raters disagreed.^b^

### Data analysis

An ecological approach to data analysis was used with school as the unit of measurement [39]. As shown in Table 1, the proportion of First Nations/Native tobacco associated with each school was estimated from a two-step process that accounted for the likelihood that First Nations/Native cigarette butts were present among the unidentifiable butts. Accordingly, the percent of cigarette butts definitively-identified as First Nations/Native was first calculated. Next, based on the assumption that the unidentifiable butts included First Nations/Native tobacco in a similar proportion to this initial calculation, the number of unidentifiable butts assumed to be First Nations/Native was calculated. This number was added to the number of butts definitively identified as First Nations/Native and used to produce a final total percent of First Nations/Native cigarette butts collected at the institution. Final values derived from the two-part calculation were used for all analyses.

**Table 1:** Percent of First Nations/Native cigarette butts by school

**School**	**Total number of butts collected**	**Butts definitively-identified as First Nations/Native**		**Final calculation of amount of First Nations/Native tobacco**	
		***n***_**identified**_	***%***	***n***_**identified**_ **+** ***n***_**assumed**_^**b**^	***%***
1^a^	1,249	11	0.88	19.9	1.60
2^a^	1,710	33	1.93	50.9	2.97
3^a^	2,440	48	1.97	77.6	3.18
4	1,662	53	3.19	69.0	4.15
5	1,203	40	3.33	56.6	4.70
6	2,046	103	5.03	115.3	5.64
7	502	27	5.38	30.7	6.12
8	916	40	4.37	56.2	6.14
9	1,621	80	4.94	115.0	7.10
10	766	53	6.92	55.5	7.24
11	848	52	6.13	77.3	9.12
12^a^	3,198	175	5.47	315.2	9.86
13	2,191	182	8.31	226.4	10.33
14	1,144	84	7.34	118.8	10.39
15	1,920	229	11.93	270.3	14.08
16	1,766	225	12.74	275.8	15.62
17	1,357	181	13.34	258.4	19.04
18	740	113	15.27	144.5	19.52
19	975	151	15.49	194.4	19.93
20^a^	810	94	11.60	162.0	20.00
21	422	75	17.77	98.1	23.25
22	1,638	375	22.89	411.6	25.13
23	2,122	649	30.58	786.9	37.08
24	1,765	562	31.84	679.2	38.48
25	1,344	447	33.26	527.5	39.25

^a^At these schools, more than half of the butts collected were unidentifiable; at the remaining 20 schools, the proportion of unidentifiable butts ranged from 5%-48%, *M* = 27%, *Mdn* = 25%.

^b^*n*_assumed_ = (number of unidentifiable butts)*(percent of butts definitively identifiable as First Nations/Native tobacco [as shown in column 4]).

Difference in the volume of First Nation/Native tobacco observed for colleges versus universities was assessed using a *t*-test. To manage the small cell sizes for the analysis comparing regions, a Kruskal-Wallis test (with post hoc Mann-Whitney *U* tests) was used.

## Results

A total of 36,355 cigarette butts were collected from the 25 post-secondary institutions. Table 1 shows the proportion of First Nations/Native tobacco among the butts collected at each school. The overall proportion of First Nations/Native butts across all schools was 14.40% (95% CIs = 9.75, 19.04). Of the 130 different cigarette brands identified, 23 were First Nations/Native (see Table 2). As presented in Table 3, differences in the volume of First Nations/Native tobacco observed on college and university campuses did not reach significance. Across the seven identified geographic regions of the province, the amount of First Nations/Native tobacco was significantly greater on campuses in the North region compared to campuses in the Central South, Southwest, East and Toronto regions.

**Table 2:** Classification of cigarette butts identified as First Nations/Native tobacco

**Category**	**Descriptive characteristics on butts**
Known First Nations/Native brands	“DK’s”, “Play Fares”, “Putters”, “KMT”, “Menage”, “Sago”
Brand names not identifiable as domestic or international legally-manufactured cigarettes	“BWE”, “CANADIAN”, “Laurel”, “NF”, “Raison Detre”, “RYG”
No visible branding, logos or other defining marks	brown filter no markings, white filter no markings, double gold stripes, double silver stripes, thick gold band, thick green band, pink stripes, blue stripe, gold stripe, green stripe, red stripe

**Table 3:** Percent of First Nations/Native cigarette butts by type of institution and geographic region

**Groups**	**Amount of First Nations/Native tobacco**	
	**Overall %**	**95% CI**
Type of Institution^a^		
College (*n* = 13)	16.75	9.65, 23.84
University (*n* = 12)	11.85	5.13, 18.57
Geographic Region^b^		
North (*n* = 5)	29.96	14.29, 45.63
Central West (*n* = 2)	19.43	−29.03, 67.90
Central East (*n* = 2)	14.53	−54.19, 83.24
Central South (*n* = 4)	10.27	−0.32, 20.87
East (*n* = 4)	9.82	−2.98, 22.62
South West (*n* = 4)	9.53	−2.96, 22.02
Toronto (*n* = 4)	5.92	4.24, 7.60

^a^*t*(23) = 1.19, *p* = .286, two tailed. ^b^*Kruskal-Wallis χ*^*2*^(6, *N* = 25) = 11.84, *p* = .066. Significantly (*p* < .05) higher amounts of First Nations/Native tobacco were observed for North compared to Central South, Southwest, East, and Toronto regions.

## Discussion

Just over 14% of the cigarette butts collected from a sample of 25 Ontario college and university campuses were First Nations/Native tobacco. Quantities of First Nations/Native tobacco found on campuses surveyed ranged from 2% to 39%. Based on the assumption that most of this First Nations/Native tobacco is being consumed illicitly [14,37], these findings suggest a fairly substantial level of contraband tobacco use among young adult, Ontario post-secondary students. While attempts to assess the validity of the current findings by comparing them to previous studies are confounded by the different age cohorts studied (i.e., youth vs. young adults) and differences in methodologies (i.e., self-report vs. collection of butts), there are some similarities. Like this study, studies of cigarette butts discarded on Ontario high school properties have shown highly variable proportions of contraband tobacco across sites (ranging from 13% to 39%) [28-30]. Similar to the overall proportion of contraband observed here (i.e., 14%), self-reports of youth [10,11,31] and adults [11,13] suggest that some 10% to 20% of tobacco consumed is contraband. In this context, the results of the current study can probably be regarded as a credible estimate of the volume of contraband tobacco being consumed by young adult smokers on college and university campuses in Ontario.

Regional variations in contraband use were observed in this study. Specifically, the highest proportion of contraband was found among institutions located in the Northern, primarily rural, region of Ontario (30%). The lowest proportion occurred among institutions in the urban, Toronto region (6%). Given that Aboriginals comprise about 2% of Ontario’s overall population, but as much as 10% of the population in the northern communities where post-secondary institutions are located [40,41], it may be that some of the First Nations/Native butts collected from campuses in the Northern region represent tobacco legally purchased by Aboriginal students who live and attend school there. While this possibility presumes that schools in northern Ontario, like the communities themselves, have a higher percentage of Native students, census data actually show lower post-secondary educational participation among Native people residing in northern Ontario compared to those residing elsewhere in the province [40]. It seems plausible to conclude, therefore, that the butts collected from northern Ontario schools do actually represent contraband tobacco.^c^

Results showing more contraband tobacco in the Northern region compared to most other geographic regions mirrors results reported by Luk et al. [13] who noted that adult smokers residing in northern Ontario were significantly more likely than those residing elsewhere in Ontario to report “usual” and “recent” purchasing of tobacco (usually First Nations/Native tobacco) on Native reserves. In Canada and elsewhere, individuals from more socially-deprived economically-depressed, rural/remote areas tend to bear a disproportionate burden of tobacco-related morbidity and mortality [3,19,22,26,27,32,43]. The greater volume of cheap contraband tobacco found for Ontario’s rural, socioecomically-stressed Northern region is consistent with this pattern, and also quite troubling given its appearance on post-secondary campuses. Higher education is generally viewed as health-protective, with lower rates of smoking uptake, less frequent smoking, and earlier cessation of any tobacco use among educated individuals. The widespread use of cheap contraband tobacco on northern Ontario post-secondary campuses suggests that education may not mitigate sociogeographic risk factors that contribute to persistent social inequities in the tobacco-related health burden. Similar findings have been reported for European [26,43], Australian [23], and American [44] samples.

The proportion of First Nations/Native tobacco was found to be slightly, but not significantly, higher on college campuses (17%) compared to university campuses (12%). Potentially higher volumes of contraband tobacco on college campuses may reflect differences in sociodemographic characteristics and smoking patterns of college and university students. There is evidence, for example, that heavier tobacco consumption is associated with greater use of contraband tobacco among youth and adults [10-13]. Similarly, lower socioeconomic status is generally related to greater likelihood of purchasing cheaper (contraband) cigarettes [13,19,27,45]. Given that family income is typically lower among college than university students [46], and rates of tobacco consumption are typically higher [47], it is not unexpected to find a higher proportion of First Nations/Native tobacco on college than university campuses.

### Limitations

While unobtrusive collection of discarded cigarette butts on campus grounds has the advantage of overcoming potential under-estimation of contraband use that may occur when participants are asked to self-report an illegal behaviour, there are some limitations to this approach. First, this approach reveals the proportion of cigarettes that are contraband, but not the proportion of individuals using contraband tobacco. Similarly, identification of contraband tobacco is restricted to what can be determined from visual inspection. Some forms of contraband tobacco (e.g., smuggled brand-name cigarettes and counterfeit cigarettes) cannot be readily identified through the process of examining discarded cigarette butts. First Nations/Native tobacco cigarette butts can be reliably identified, but are contraband only if the manufacture or sale of these cigarettes violates government regulations—contingencies that could be assumed but not verified in this observational study. These limitations must be weighed against evidence that smuggled brand-name and counterfeit cigarettes make up a very small proportion of contraband tobacco in Ontario [14,37], and the general consensus in the Canadian literature [10,12,13,31] that large proportions of First Nations/Native tobacco are produced or consumed illicitly and can reasonably be assumed to represent contraband tobacco. Finally, the exclusive use of First Nations/Native tobacco to represent contraband tobacco should not be seen as a commentary on First Nations manufacturers of tobacco, the legality of production, packaging and sales of First Nations/Native tobacco, or the actions and attitudes of First Nations people in Ontario.

### Implications

Results of the current study showing geographically widespread and relatively high levels of use of First Nations/Native (contraband) tobacco by young adults suggest that public health initiatives to reduce smoking among young adults need to address this group’s use of contraband as well as commercial tobacco. Taxation has been a powerful public health strategy for reducing tobacco use among all age groups, but especially younger smokers who are price-sensitive [3,4,7,8,12,13,35]. In the absence of contraband tobacco, higher taxes on regulated tobacco discourage smoking uptake and encourage cessation of smoking [4,5,7,8]. In the presence of contraband tobacco, however, higher taxes can encourage individuals to seek out cheap contraband tobacco [22,27,32,45,48].

The likely ineffectiveness of taxation strategies in the current market indicates the need for other anti-contraband strategies. Enforcement of laws related to international movement of dutiable, taxable, controlled goods, and to the manufacture and distribution of First Nations/Native and other tobacco products can be effective strategies to deal with tobacco growers, cigarette manufacturers and distributors, and retailers who shape and sustain the contraband market [13,14,49]. In Canada, law enforcement in these critical areas generally falls to the federal government. In the case of First Nations/Native tobacco, however, enforcement strategies to reduce the availability of contraband tobacco necessitates cooperation among federal, provincial and municipal law enforcement officers, as well as First Nations’ own police services. To this end, federal initiatives including Public Safety Canada’s *First Nations Organized Crime Initiative*[50], and the RCMP-led *Combined Forces Special Enforcement Unit, Contraband Tobacco Initiative*[51] have begun to disrupt the flow of contraband tobacco through successful inter-governmental, multi-agency collaboration [37]. As the current study and others [13,29,30] show, it will be necessary for these nationally-led initiatives to respond to regional variations between and within provinces to effectively reduce availability of cheap contraband tobacco and impact tobacco use among smokers of all ages.

Law enforcement can also target consumers of contraband tobacco, however its impact in this regard may be limited. Penalizing individual smokers for purchasing illicit cigarettes may do little to contain the contraband market [48]. Public education, on the other hand, may be a more viable way to address the consumer side of the contraband market [9,23,48]. In this regard, development of coherent, action-oriented public education messages that specifically motivate and support young adult smokers to avoid contraband tobacco will require attention to several challenges. Most notably, the message that First Nations/Native tobacco is being manufactured or sold in a manner that makes it illegal must not support negative stereotypes of First Nations people. In fairness to all residents of Ontario and Canada, this potential consequence is one that should be anticipated and avoided. Public education campaigns describing the illicit nature of non-Aboriginals’ use of First Nations/Native tobacco must also avoid the unintended consequence of raising young adult smokers’ awareness of this cheap tobacco supply to the point of promoting rather than discouraging its use. Similarly, because efforts to inform the public that inexpensive contraband tobacco does not necessarily adhere to government health and safety guidelines can create the impression that commercial cigarettes are healthier or safer, young adults—like all smokers—must be informed that no cigarettes are good for health.

Public education messages presenting the use of contraband tobacco as unlawful based on tax evasion must account for the likelihood that most people, including young adults, seem unconcerned with or unaware of the illegality of not paying taxes on cigarettes [27,31,32,48]. Describing how this personal act of unlawfully purchasing tobacco can undermine global health objectives, reduce tax revenues that support education and social programs, contribute to the proliferation of organized crime, and weaken legitimate local economies will be effective only if the messages resonate with young adults. Thus, reminding the public that decreases in government revenues from tobacco taxes translate into less funding for the health care system and could ultimately affect them personally may be more effective for adults than young adults who are typically unconcerned with personal access to or public expenses of healthcare. Likewise, messages explaining that cheap tobacco increases smoking uptake and escalation among youth may dissuade adults—especially those with children—from purchasing First Nations/Native tobacco, but have limited impact on young adults. Issues such as social equity and responsibility, funding for public education, economic justice, and control of organized crime may be more relevant to young adults. Similarly, because young adults may be unaware that purchasing First Nations/Native tobacco is in fact illegal, campaigns that convey this message, while highlighting the compelling social costs of a contraband market and avoiding the pitfall of re-legitimizing commercial tobacco, may deter young adults from using contraband tobacco. In any case, careful age-tailoring of messages will be required.

## Conclusions

Overall, as a first step in determining the extent to which contraband tobacco is used by young adults, the current study revealed highly variable, but substantial levels of contraband tobacco use among young adult, Ontario post-secondary students. A more fine-grained analysis of the types of contraband tobacco being used by young adults would inform the optimal mix of anti-contraband enforcement and public education strategies. Additional research is needed to determine characteristics of young adult contraband tobacco users and the relationships between contraband tobacco use and young adults’ smoking and quitting behaviours. This understanding would support successful development and implementation of public health initiatives to reduce young adults’ use of contraband tobacco and potentially reduce initiation, escalation and continuation of smoking in this cohort.

## Endnotes

^a^ At one college, an oversight resulted in only 3 (vs. 4) containers being collected.

^b^ It should be noted that visual inspection of cigarette butts did not allow for reliable, fine-grained identification of specific types of contraband (e.g., illegally-manufactured tobacco; smuggled tobacco; illegally-diverted tax-exempt tobacco). Among unbranded cigarettes, for example, it was not possible by visual inspection to differentiate between cigarettes illegally-manufactured in Canada and cigarettes manufactured in the U.S. and smuggled into Canada. Similarly, while the illicit nature of First Nations/Native brand name cigarettes is generally attributable to diversion of these tax-exempt cigarettes to non-Aboriginal populations, these cigarettes can also be produced illegally as evidenced by an Ontario’s Auditor General report indicating one single manufacturer of First Nations/Native tobacco was producing more than double the number of tax-exempt cigarettes legally allowable for all manufacturing companies in the province [38]. Visual inspection cannot detect this difference.

^c^ School-level enrolment data for First Nations students are not available for the majority of post-secondary institutions, nor is this information uniformly available or plausibly extractable from alternate data sources (see: http://www.tcu.gov.on.ca/pepg/publications/framework.html, *pp* 19–20) [42].

## Competing interests

The authors declare that they have no competing interests.

## Authors’ contributions

This study was conducted as MB’s Masters thesis research under the supervision of KL. Both authors contributed to the conception and design of the study. MB collected, analyzed and interpreted the data. KL drafted the manuscript. Both authors read and approved the final manuscript.

## Pre-publication history

The pre-publication history for this paper can be accessed here:

http://www.biomedcentral.com/1471-2458/13/335/prepub
